# Predictive CT features for the diagnosis of primary pulmonary mucoepidermoid carcinoma: comparison with squamous cell carcinomas and adenocarcinomas

**DOI:** 10.1186/s40644-020-00375-2

**Published:** 2021-01-06

**Authors:** Xiaohua Ban, Xinping Shen, Huijun Hu, Rong Zhang, Chuanmiao Xie, Xiaohui Duan, Cuiping Zhou

**Affiliations:** 1 Department of Medical Imaging Center, State Key Laboratory of Oncology in South China, Collaborative Innovation Center for Cancer Medicine, Sun Yat-sen University Cancer Center, 651 Dongfeng Road East, Guangzhou, Guangdong 510060 People’s Republic of China; 2 Department of Radiology, The University of Hong Kong-Shenzhen Hospital, No.1, Haiyuan Road Futian District, Shenzhen, 518000 People’s Republic of China; 3 Department of Radiology, Guangdong Provincial Key Laboratory of Malignant Tumour Epigenetics and Gene Regulation, Sun Yat-Sen Memorial Hospital, Sun Yat-Sen University, No. 107 Yanjiang Road West, Guangzhou, Guangdong 510120 People’s Republic of China

**Keywords:** Mucoepidermoid carcinomas, Primary, Predictive, Diagnosis, Computed tomography

## Abstract

**Background:**

To determine the predictive CT imaging features for diagnosis in patients with primary pulmonary mucoepidermoid carcinomas (PMECs).

**Materials and methods:**

CT imaging features of 37 patients with primary PMECs, 76 with squamous cell carcinomas (SCCs) and 78 with adenocarcinomas were retrospectively reviewed. The difference of CT features among the PMECs, SCCs and adenocarcinomas was analyzed using univariate analysis, followed by multinomial logistic regression and receiver operating characteristic (ROC) curve analysis.

**Results:**

CT imaging features including tumor size, location, margin, shape, necrosis and degree of enhancement were significant different among the PMECs, SCCs and adenocarcinomas, as determined by univariate analysis (*P* < 0.05). Only lesion location, shape, margin and degree of enhancement remained independent factors in multinomial logistic regression analysis. ROC curve analysis showed that the area under curve of the obtained multinomial logistic regression model was 0.805 (95%CI: 0.704–0.906).

**Conclusion:**

The prediction model derived from location, margin, shape and degree of enhancement can be used for preoperative diagnosis of PMECs.

## Background

Primary pulmonary mucoepidermoid carcinoma (PMEC) is a rare malignant neoplasm of the lung and accounts for approximately 0.1–0.2% of all lung malignancies [1–5], arising from the minor salivary glands of the tracheobronchial tree [6]. Histologically, PMEC consists of mucous-forming, epidermoid and intermediate cells [3, 7]. It has been reported that PMECs are usually well differentiated [8], and associated with weak invasive and good prognosis, compared with the most common primary pulmonary malignancies such as squamous cell carcinomas (SCCs) and adenocarcinomas [9, 10]. Therefore, distinguishing PMECs from primary SCCs and adenocarcinomas has potential prognostic and therapeutic implications. At present, the clinical and pathological features, management of PMECs have been well documented [1–8]. Moreover, some imaging findings of PMECs have been described [10–12]. However, little is known about the most important imaging features for differential diagnosis of PMECs from pulmonary SCCs and adenocarcinomas.

In this study, we retrospectively reviewed the CT findings of PMECs, SCCs and adenocarcinomas in order to identify the independent predictive radiological features for the diagnosis of PMECs.

## Materials and methods

### Patients

Between July 2012 and March 2019, 37 patients with pathologically proven PMECs were retrospectively reviewed. In addition, 76 patients with SCCs and 78 patients with adenocarcinomas from September 2018 to July 2019 were included in order to analyze the characteristic CT features of PMECs. Patients were included if they were pathologically confirmed, and chest CT examination before treatment. Patients with the same tumor in any other organ or have previous history of the same tumor were excluded. This study was approved by the Institutional Review Board of every participating center, and patient informed consent was waived for this type of review.

Clinical data including age, sex, and clinical presentation were reviewed. The main symptoms of three diseases included cough, phlegm, pectoralgia, fever and asthma.

### CT imaging

All patients had chest CT imaging before treatment. CT imaging was performed in 88 patients using a 64-slice spiral CT (Toshiba Aquilion 64, Toshiba Medical Systems, Japan), and in the remaining 103 patients using a 64-slice spiral CT (LightSpeed VCT, GE Medical Systems, Milwaukee, WI, USA). The main imaging parameters were 120 kVp, 150–300 mA of automatic adjustment, a pitch of 0.982. Axial and coronal multiplanar reconstructions (MPR) images with 2–5 mm thick were obtained with lung or mediastinum kernels. After acquisition of unenhanced images, contrast-enhanced images were obtained after a bolus intravenous injection of 1.5 ml/kg contrast media (Iopamiro 370; Bracco S.P.A., Milan, Italy), with a rate of 3 ml/s.

### Imaging analysis

CT data were reviewed on PACS system for all patients by two experienced radiologists in consensus (C.Z., with more than 12 years of experience with diagnostic imaging, and X.D., with 10 years of experience with diagnostic imaging). CT imaging features including primary tumor size, location, margin, shape, calcification, necrosis and degree of enhancement were determined. Tumor size was measured in maximal dimension on the transverse plane. The location of the lesions was classified as central type which is in main bronchi and trachea, hilar type which is in lobar or segment bronchi or the right middle bronchus, and peripheral type which is in small bronchi. The margin of tumor is considered as well-defined or ill-defined. The shape of the lesion was classified as irregular or regular. The regular shape was defined as round/ovoid, and irregular was lobulated. Degrees of enhancement were graded as mild, moderate and marked. Comparing the enhanced and plain CT, degree of enhancement was defined as mild if the increased CT value after enhancement is lower than or equal to 20 HU, as moderate if greater than 20HU but great less than or equal to 40 HU, and as marked if greater than 40 HU. Intra-tumor necrosis was discerned on contrast-enhanced CT images and tumor calcification was identified on unenhanced CT images.

### Statistical analysis

The CT imaging features for statistical analysis were categorized as follows: lesion location (central, hilar or peripheral), margin (well-defined or ill-defined), shape (irregular or regular), necrosis (presence or absence), calcification (presence or absence), degree of contrast enhancement (mild, moderate or marked). Univariate analysis was applied to compare the frequency of these imaging findings among PMECs, SCCs and adenocarcinomas by using χ^2^ test or Fisher’s Exact Test, and compare lesion size of three groups by variance analysis. A multinomial logistic regression analysis was subsequently developed to determine the association between the three groups and radiologic variables. Variables with *P* value less than 0.05 as determined by univariate analysis were further chosen as the independent predictor for diagnosis of PMECs in the multivariate logistic regression analysis. A two-side *P* value of less than 0.05 was considered statistically significant. Furthermore, receiver operating characteristic (ROC) curve analysis was used to evaluate the obtained multinomial logistic regression model. All statistical tests were performed by using software SPSS (version 22.0, Chicago, IL, USA).

## Results

### Clinical findings

In cases of PEMCs, there were 15 women (40.5%) and 22 men (59.5%), aged from 10 to 77 years with a mean of 44.95 years ±19.26. In cases of SCCs, there were 7 women (9.2%) and 69 men (90.8%), aged from 36 to 83 years with a mean of 63.11 years±7.72. In cases of adenocarcinomas, there were 31 women (39.7%) and 47 men (60.3%), aged from 37 to 93 years with a mean of 61.83 years±10.28.

### CT imaging features

**Fig. 1:**
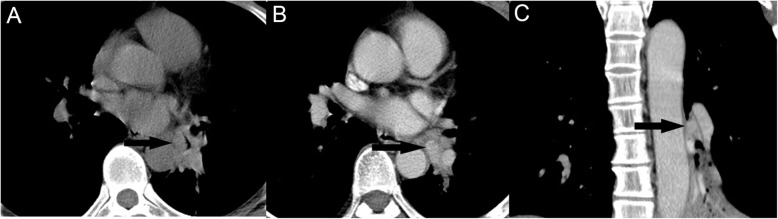
A PMEC with regular shape and well-defined margin with hilar type. Axial non-contrast CT image (**a**) shows a isodense mass (arrows) in left inferior lobar bronchus. Contrast-enhanced CT image (**b**, **c**) shows tumor with marked enhancement without necrosis (arrows)

**Fig. 2:**
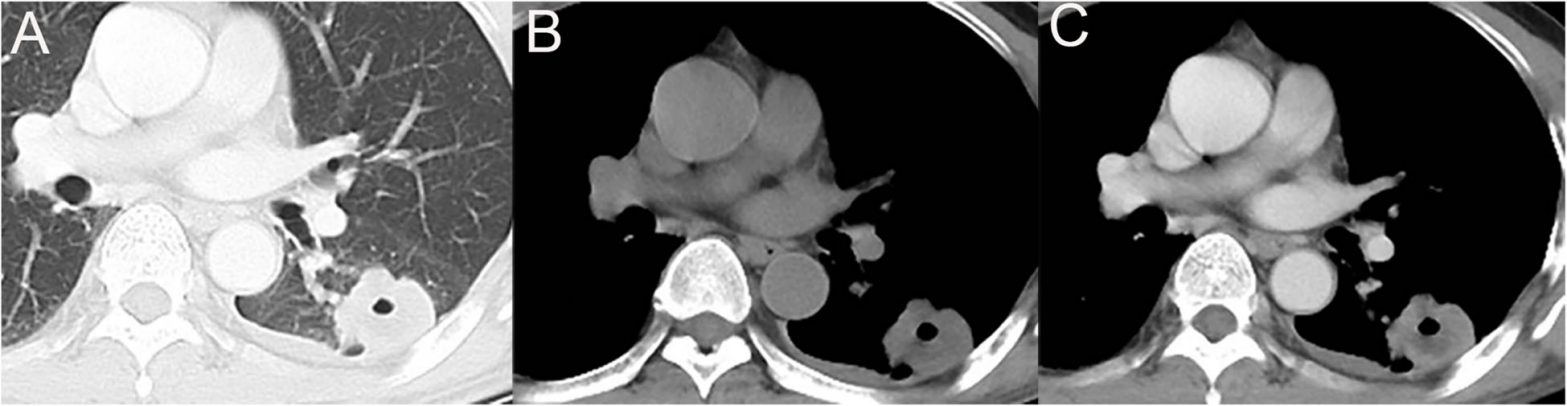
A PMEC with irregular shape and ill-defined margin with peripheral type. Axial non-contrast CT image (**a**,**b**) shows a hypodense mass (arrows) in left inferior lobar dorsal segment. Contrast-enhanced CT image (**c**) shows tumor with moderate enhancement and necrosis (arrows)

The main CT characteristics of three groups are summarized in Table 1. In cases of PMECs, the size of the lesions ranged from 0.6 to 6.5 cm, with a mean 2.5 cm. Tumors were central type in 4 patients (10.8%), hilar type in 29 patients (78.4%) (Fig. 1) and peripheral type in 4 patients (10.8%) (Fig. 2). The margin of the PMECs was well-defined (Fig. 1) in 27 patients (73.0%) and ill-defined in the remaining 10 patients (27.0%). Lesions were irregular in 6 patients (16.2%), while regular in other 31 patients (83.8%) (Fig. 1). PMECs showed mild enhancement in 2 patients (5.4%), moderate enhancement in 10 patients (27%) and marked enhancement in 25 patients (67.6%) (Fig. 1) on contrast-enhanced CT images. Necrosis was observed in 3 patients (8.1%) with PMECs, 30 patients (39.5%) with SCCs, and 18 patients (23.7%) with adenocarcinomas. Tumor calcification was found in 6 patients with PMECs, 16 patients with SCCs, and 10 patients with adenocarcinomas. In cases of SCCs, tumors were central type in 2 patients (2.6%), hilar type in 33 patients (43.4%) and peripheral type in 41 patients (53.9%). The SCCs were irregular in 46 patients (60.5%), and regular in 30 patients (39.5%). There are 33 SCCs (43.4%) with mild enhancement, 32 (42.1%) with moderate enhancement and 11 (14.5%) with marked enhancement on contrast-enhanced CT images. In cases of adenocarcinoma, tumors were central type in 1 patient (9%), hilar type in 5 patients (78.4%) and peripheral type in 71 patients (91%). The adenocarcinomas presented irregular in 23 patients (29.5%), and regular in 55 patients (70.5%). Adenocarcinomas showed mild enhancement in 27 patients (34.5%), moderate enhancement in 31 patients (39.1%), and marked enhancement in 20 patients (25.6%) on contrast-enhanced CT images.

**Table 1:** CT features of three groups of patients

Characteristics	PMECs		SCCs		Adenocarcinomas	
	No. of patients	%	No. of patients	%	No. of patients	%
Tumor size(cm)	2.5 ± 0.2		4.9 ± 0.2		3.9 ± 0.2	
Location						
Central type	4	10.8	2	2.6	2	2.6
Hilar type	29	78.4	33	43.4	5	6.4
Peripheral type	4	10.8	41	53.9	71	91.0
Margin						
Well-defined	27	73.0	17	22.4	32	41.0
Ill-defined	10	27.0	59	77.6	46	59.0
Tumor shape						
Irregular	6	16.2	46	60.5	23	29.5
Regular	31	83.8	30	39.5	55	70.5
Necrosis						
Present	3	8.1	30	39.5	18	23.7
Absent	34	91.9	46	60.5	60	76.9
Calcification						
Present	6	16.2	16	21.1	10	12.8
Absent	31	83.8	60	78.9	68	87.2
Enhancement degree						
Marked	25	67.6	11	14.5	20	25.6
Moderate	10	27.0	32	42.1	31	39.7
Mild	2	5.4	33	43.4	27	34.6

### Predictive value of imaging features for diagnosis of PMEC

**Fig. 3:**
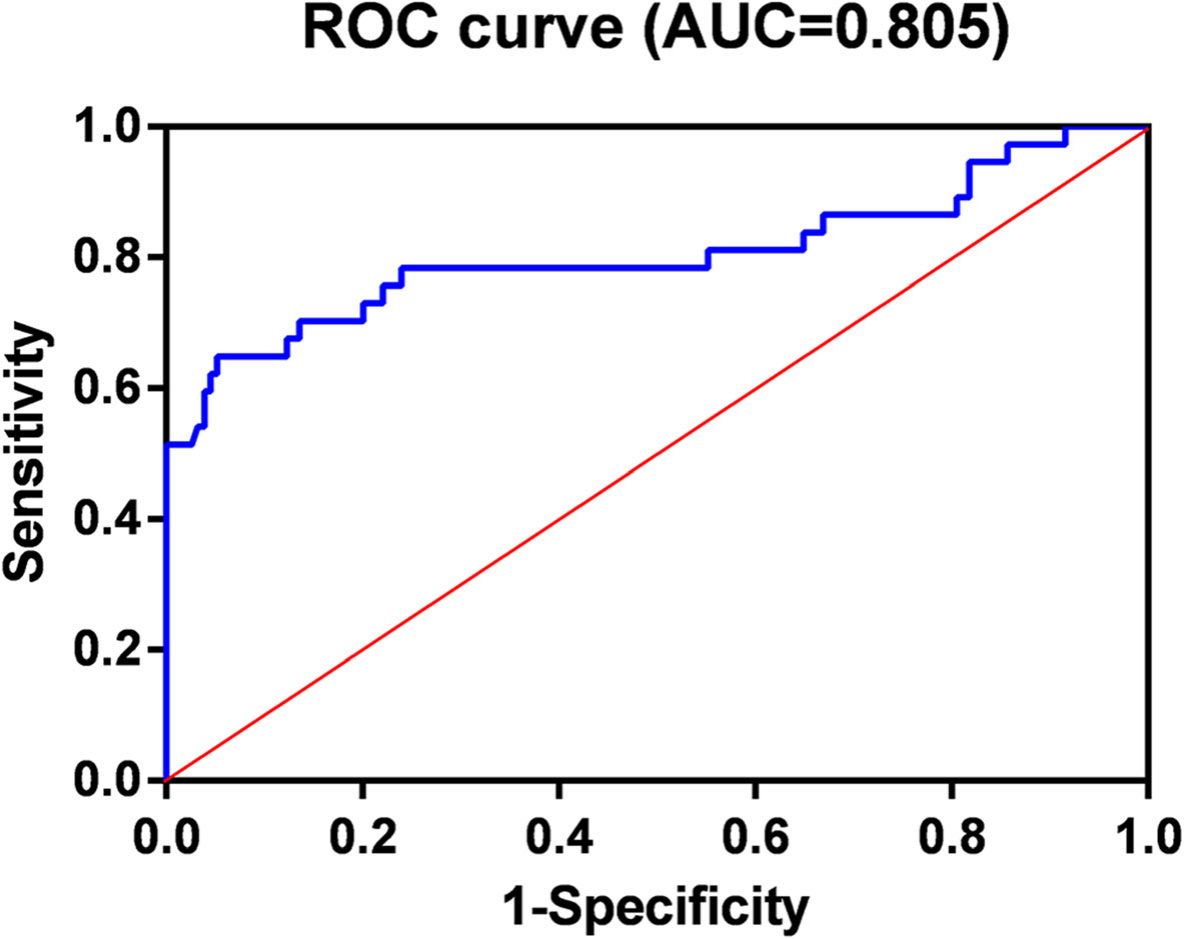
Graph shows the receiver operating characteristic (ROC) curve of area multinomial logistic regression model. The area under the curve (AUC) was 0.805

In univariate analysis, tumor size, location, margin, shape, necrosis and enhancement degree were found to be sigificant different among the three groups of tumors (*P* < 0.05) (Table 2). Multinomial logistic regression analysis showed that location, margin, shape, and enhancement degree category of primary tumor remained independent predictors for differential diagnosis of PMECs and SCCs (Table 3). Moreover, location, margin, and shape were predictors for differential diagnosis of PMECs and adenocarcinomas (Table 4). The primary tumors in lung with central type (OR = 177.326) or hilar type (OR = 56.484), well-defined margin (OR = 6.738), regular shape (OR = 16.560), and/or marked enhancement (OR = 10.618) are more likely to be PMECs than SCCs (*P* < 0.05). Meanwhile, the primary tumors in lung with central type (OR = 185.586) or hilar type (OR = 518.164), well-defined margin (OR = 7.283), regular shape (OR = 9.279) are more likely to be PMECs than adenocarcinomas (*P* < 0.05). Further ROC curve analysis showed that the area under curve (AUC) of the obtained logistic regression model was 0.805 (95% CI: 0.704–0.906) (Fig. 3), indicated that the model was a reasonable predictor for differential diagnosis of PMECs from SCCs and adenocarcinoma.

**Table 2:** Univariate analyses of radiologic factors

Factor	Category	No. of PMECs, SCCs vs. adenocarcinomas
Tumor size	Adenocarcinomas	3.9 ± 0.2*
	SCCs	4.9 ± 0.2**
	PMECs	2.5 ± 0.2
Location	Peripheral type	4:41:32**
	Hilar type	29:33:5
	Central type	4:2:1
Margin	Ill-defined	10:59:46**
	Well-defined	27:17:32
Tumor shape	Irregular	6:46:23**
	Regular	31:30:55
Necrosis	Present	3:30:18*
	Absent	34:46:60
Calcification	Present	6:16:10
	Absent	31:60:68
Enhancement degree	Marked	25:11:20**
	Moderate	10:32:31
	Mild	2:33:27

*P < 0.05; ** *P* < 0.01

**Table 3:** Mutiple logistic regression analysis of PMECs and SCCs

Factors	Category	β value	*P* value	OR (95% CI)
Tumor size		−0.067	0.234	0.935 (0.591,1.479)
Location	Central type	5.178	0.001	177.326 (7.422,4236.506)
	Hilar type	4.034	< 0.001	56.484 (8.301, 384.370)
Margin	Well-defined	1.908	0.36	6.738 (1.130, 40.178)
Tumor shape	Regular	2.807	0.001	16.560 (3.275,83.730)
Necrosis	Present	1.352	0.161	3.864 (0.583,25.630)
Enhancement degree	Marked	2.363	0.022	10.618 (1.396,80.730)
	Moderate	1.998	0.053	7.376 (0.971,56.005)

**Table 4:** Mutiple logistic regression analysis of PMECs and adenocarcinomas

Factors	Category	β value	*P* value	OR (95% CI)
Tumor size		−0.036	0.883	0.965 (0.596,1.562)
Location	Central type	5.224	0.001	185.586 (9.724,3541.880)
	Hilar type	6.250	< 0.001	518.164 (66.265,4051.846)
Margin	Well-defined	1.985	0.039	7.283 (1.101, 48.174)
Tumor shape	Regular	2.228	0.011	9.279 (1.655,52.024)
Necrosis	Present	0.511	0.617	1.667 (0.225,12.346)
Enhancement degree	Marked	1.835	0.085	6.267 (0.777,50.577)
	Moderate	1.932	0.069	6.904 (0.861, 55.328)

## Discussion

Primary PMECs are rare and still a challenge for diagnosis. Preoperative identification of patients with PMECs is important for the assessment of clinical outcome and the choice of treatment [13, 14]. Herein, we analyzed and compared the CT features of PMECs with the common type of lung cancers (SCCs and adenocarcinomas). Our study demonstrated that a prediction model derived from location, margin, shape and enhancement degree category determined with preoperative CT imaging features was a useful tool for distinguishing PMECs from the common type of lung cancers. The AUC of the obtained logistic regression model was 0.805 (95% CI: 0.704–0.906), indicates that this model has discriminative ability to identify the patients with PMECs.

Clinically, PMECs tended to affect young adults [15], which was similar to our results with a mean of 44.95 years. The gender distribution of PMECs in our study was 22:15, with slight male predilection, which was consistent with previous report [16]. While, SCCs commonnly occur in older male, with a mean of 63.11 years in our series. Adenocarcinomas also had slight male prediletion, but normally happens in older peoples with a mean of 61.83 years.

PMECs are commonly found in the segmental or lobar bronchi [8, 17–19]. The main radiographic features were well demarcated masses with smooth borders, and round shape [15, 16, 20, 21]. In our series, PMECs were hilar type in 78.4% patients, well-defined in 73.0% patients, and regular in 83.8% patients. Furthermore, PMECs usually showed marked heterogeneous contrast enhancement on CT images [15, 20, 21]. However, the results from different studies are not consistent. Cheng et al. reported that MSCT revealed a mass with mild enhancement, as manifested by calcification and visible mucus lakes, which may be suggestive of PMECs [11]. Kim et al. also reported that punctate calcification within the tumor was seen in 50% patients [18]. Our results showed that PMECs were moderate enhancement in 27% patients and marked enhancement in 67.6% patients. Tumor calcification was just found in 6 patients (16.2%) with PMECs. Necrosis was only observed in 3 patients (8.1%) with PMECs. What’s more, tumors with central or hilar type of location, well-defined margin, regular shape, absent necrosis, mark or moderate enhancement degree were associated with PMEC in univariate analysis.

To improve diagnosis, we used multinomial logistic regression analysis to identify the predictive radiological features for the diagnosis of PMECs. In our study, PMECs were central and hilar type in 89.2%, SCCs were central and hilar in 46.1% patients, and adenocarcinomas were central and hilar just in 9% patients. In multinomial logistic regression analysis, location of lung cancer is an independent feature for differential diagnosis. Primary lung cancer with central type of location are more likely to be PMEC than SCC (OR = 177.326) or adenocarcinoma (OR = 185.586), and the tumor with hilar type of location is also more likely to be PMEC than SCC (OR = 56.484) or adenocarcinoma (OR = 518.164). PMECs are usually arises from large airway, including the trachea and the main or lobar bronchi [8, 22]. However, adenocarcinomas tend to occur in peripheral lung. Meanwhile, the well-defined primary lung cancer is more possible to be PMEC than SCC (OR = 6.738) or adenocarcinoma (OR = 7.283). Moreover, the primary tumor in lung with regular shape is more likely to be PMEC than SCC (OR = 16.560) or adenocarcinoma (OR = 9.279). PMEC appears as a smoothly oval or lobulated airway mass at CT, which adapts to the branching features of the airway [18, 19]. In addition, the primary tumor in lung with marked enhancement (OR = 10.618) are more likely to be PMEC than SCC. Mucoepidermoid carcinoma of the bronchus is often visualized as marked heterogeneous contrast enhancement on high-resolution CT images because of the presence of abundant microvessels on microscopic examination [20].

Our study has several limitations. First, as a retrospectively study using CT data from different hospitals over different periods of years, it would inevitably suffer from some degree of selection bias and various CT facilities and scans parameters. However, all patients in our series had slice thicknesses of CT images less than 5 mm, which may be acceptable for imaging analysis. Second, the number of patients in this study was limited because of PMECs are rare and the follow-up period is just 7 years. Third, no state-of-art CT was used in our study, such as dual-source or spectral CT. CT spectral quantitative parameters are valuable in evaluating histological types of lung cancers, and it is maybe even possible to correctly determine the lesion density before contrast enhancement [23]. Future prospective studies with a large sample size or using state-of-art CT may provide additional information for the diagnosis of PMEC.

## Conclusion

In conclusion, our study results suggest that CT features including location, margin, shape, and degree enhancement are predictors for diagnosis of PMEC. The primary lung tumor with central type or hilar type, well-defined margin, regular shape, and marked enhancement indicates PMEC.

## Data Availability

Not applicable.

## Abbreviations

CT: Computed tomography
PMEC: Primary pulmonary mucoepidermoid carcinoma
SCC: Squamous cell carcinoma
MPR: Multiplanar reconstructions
HU: Hounsfield unit
ROC: Receiver operating curve
OR: Odds ratio
AUC: Area under curve
CI: Confidence interval
